# Analysis of β-catenin association with obesity in African Americans with premalignant and malignant colorectal lesions

**DOI:** 10.1186/s12876-020-01412-x

**Published:** 2020-08-18

**Authors:** Babak Shokrani, Hassan Brim, Tahmineh Hydari, Ali Afsari, Edward Lee, Mehdi Nouraie, Zaki Sherif, Hassan Ashktorab

**Affiliations:** 1 Department of Medicine, Department of Pathology and Cancer Center, Howard University College of Medicine, 2041 Georgia Avenue, N.W, Washington, D.C, 20060 USA; 2 Division of Pulmonary, Allergy and Critical Care Medicine, University of Pittsburg, Pittsburg, PA USA

**Keywords:** β-Catenin, Colorectal cancer, Advanced adenoma, African Americans

## Abstract

**Background:**

African Americans (AA) are at high risk for Colorectal Cancer (CRC). Studies report a 30–60% increase in CRC risk with physical inactivity, obesity and metabolic syndrome. Activation of the WNT/β-catenin (CTNNB1) signaling pathway plays a critical role in colorectal carcinogenesis. Accumulating evidence also indicates a role of WNT-CTNNB1 signaling in obesity and metabolic diseases.

**Aim:**

To examine the association between obesity, β-Catenin expression and colonic lesions in African Americans.

**Methods:**

We reviewed the pathology records of 152 colorectal specimens from 2010 to 2012 (46 CRCs, 74 advanced adenomas and 32 normal colon tissues). Tissue Microarrays (TMA) were constructed from these samples. Immunohistochemistry (IHC) for CTNNB1 (β-Catenin; clone β-Catenin-1) was performed on the constructed TMAs. The IHC results were evaluated by 2 pathologists and the nuclear intensity staining was scored from 0 to 4. BMI, sex, age, location of the lesion and other demographic data were obtained.

**Results:**

Positive nuclear staining in normal, advanced adenoma and CRC was 0, 24 and 41%, respectively (*P* < 0.001). CRC was asso ciated with positive status for nuclear CTNNB1 intensity (adjusted OR: 3.40, 95%CI = 1.42–8.15, *P* = 0.006 for positive nuclear staining) compared to non-CRC samples (Normal or advanced adenoma). Nuclear staining percentage has a fair diagnostic ability for CRC with an AUC of 0.63 (95%CI = 0.55–0.71).

Overweight/obese patients (BMI > 25) did not show a significant difference in (*p* = 0.3) nuclear CTNNB1 staining (17% positive in normal weight vs. 27% positive in overweight/obese). The association between nuclear intensity and CRC was not different between normal and overweight patients (P for interaction = 0.6). The positive nuclear CTNNB1status in CRC stage III and IV (35% of all CRC) was not different from stage I and II (50% vs. 36%, respectively, *P* = 0.4).

**Conclusion:**

In our study, advanced adenoma and CRC were associated with activation of β-catenin in physically fit, overweight and obese patients. Thus, obesity and WNT/β-Catenin pathway seem to be independent in African American patients. WNT/β-Catenin signaling pathway has a potential to be used as an effector in colon carcinogenic transformation. Whether or not BMI is a modifier of this pathway needs to be investigated further.

## Background

Colorectal cancer (CRC) is one of the most common cancers in the industrialized world [1]. Lifestyle and epidemiological factors associated with an increased risk of CRC include physical inactivity, obesity and metabolic syndrome [2]. In the United States, approximately two-thirds of the adult population are overweight or obese, which represents a putative risk factor for multiple target organ malignancies, including CRC [3].

There is evidence to suggest that excess adiposity is associated with up to 60% greater risk of CRC compared with normal weight individuals [4], and that physical activity may decrease colorectal cancer risk [5]. Although excessive accumulation of white adipose tissue (WAT) is the key feature of adiposity, obesity is clinically defined by a BMI over 30 kg/m^2^, which does not take fat content into account. It is also known that most CRCs arise from a genetic and morphological adenoma to carcinoma transition. Also, it is widely accepted that both CRCs and colorectal adenomas (CRAs) share similar etiological causes which explains why CRAs, which are amongst the most frequent pathological findings in all CRC screening participants, are present in more than 30% of general asymptomatic populations [6]. Consequently, risk algorithms have been applied to use BMI as a predictor variable to stratify individuals according to their risk of colorectal neoplasia [7]. However, the underlying mechanisms that might explain the association and the magnitude of the connection between excess body weight and CRC remain unclear.

In the obesity-cancer relationship, multiple biological processes including insulin, insulin-like growth factor (IGF)-1, insulin resistance, sexual hormones (estrogens) and pro-inflammatory cytokines (TNF-α, IL-6 and CRP) actively participate [8]. All these elements create a favorable environment for carcinogenesis and a decrease in apoptosis.

As a separate molecular pathway, activation of the WNT signaling pathway plays a critical role in colorectal carcinogenesis [9]. WNT ligands are a family of proteins that are important for normal cell development. β-Catenin (CTNNB1) is a major mediator of the WNT pathway, that is traditionally classified into canonical (β-Catenin-dependent) and non-canonical (β-Catenin-independent). WNT canonical pathway utilizes a group of cell surface receptors called frizzled (FRZ) to activate several pathways, the most important one involving β-Catenin and APC [10]. In the absence of WNT signaling, APC causes degradation of β-Catenin, preventing its accumulation in the cytoplasm by forming a complex with β-Catenin, which leads to the phosphorylation and eventually destruction of β-Catenin by the proteasome. Signaling by WNT blocks this process, allowing β-Catenin to migrate from the cytoplasm to the nucleus. Once in the nucleus, β-Catenin up-regulates c-*MYC*, *cyclin D1*, and other genes which increase cellular proliferation [11]. Therefore, continuous WNT signaling can be seen in cells with loss of APC [12].

Metabolic syndrome-associated conditions such as obesity and type II diabetes are influenced by genetic and functional variations in the WNT signaling pathway [13]. WNT signaling, when activated, represses the terminal differentiation during adipogenesis whereby pre-adipocytes take on the characteristics of mature adipocytes. A cascade of transcriptional events like the induction of β-Catenin ensues, which in turn induces enhancer binding protein-α (CEBPA) and peroxisome proliferator-activated receptor-γ (PPARG) [14]. The excessive accumulation of WAT features adiposity but obesity does not take fat content into account [15]. Recently, genetic factors linked to fat mass and adiposity were reported to be associated with increased obesity risk [16]. In young obese individuals, whole-exome sequencing revealed rare gain-of-function mutations in *CTNNB1/*β-catenin [16]. The β-Catenin-regulated transcription of an adipocyte-derived chemokine called *serum amyloid A3* (*Saa3*) leads to the formation of a β-Catenin–TCF complex in mature adipocytes that promotes the proliferation of pre-adipocytes in WAT and thereby increases obesity and the risk for metabolic syndrome. Other data also suggest that obesity and lack of physical activity are associated with a higher risk for colorectal cancer [17, 18]. These findings have important implications especially in the obese and physically inactive African American population that may have underlying predisposing mutations to colorectal cancer [19].

The aim of this study is to assess the β-Catenin expression profile in colorectal pre-malignant and malignant lesions in correlation with obesity as determined by body mass index (BMI) or waist circumference (WC) in African American population.

## Methods

### Patients and clinical data

Colorectal tissue samples submitted to the Surgical Pathology Laboratory at Howard University Hospital from January 1, 2010 to December 31, 2012 were retrieved from the pathology archive system (PowerPath™). A total of 152 samples were included in the study consisting of tissue samples from CRC (*n* = 46), advanced adenoma (*n* = 74) and normal colon (*n* = 32). Patients’ data included age, sex, height, weight and waist circumference. Body mass index (BMI) was calculated in the study (Table 1). The protocol of this study was approved by the Howard University Institutional Review Board (IRB).

**Table 1:** Epidemiological characteristics and BMI in normal, advanced adenoma and CRC patients

	Normal *N* = 32	Advanced Adenoma *N* = 74	CRC *N* = 46	*P* value
Age, median (IQR)	63 (55–75)	61 (56–64)	68 (53–76)	0.004
Male, n (%)	15 (47)	24 (57%)	24 (48)	0.8
BMI (kg/m2), median (IQR)	26.1 (22.6–29.9)	29.5 (26.3–35.9)	29.3 (20.8–35.3)	0.3
Overweight, n (%)	15 (60)	32 (78)	20 (69)	0.3

### Tissue microarray (TMA) construction

Hematoxylin-Eosin stained slides (H&E slides) were made from paraffin-embedded blocks. The H&E slides were reviewed by two pathologists to confirm the pathological diagnosis and to mark areas of interest. Multiple areas from more than one block were marked to ensure a good representability of the sample on the TMA. Five TMA paraffin blocks, each containing 75 cores of 1 mm in diameter each and 0.5 mm distance from each other, were constructed. Tissue-specific and organ system controls were included in each TMA.

### Immunohistochemical (IHC) analysis of CTNNB1 (β-catenin)

**Fig. 1:**
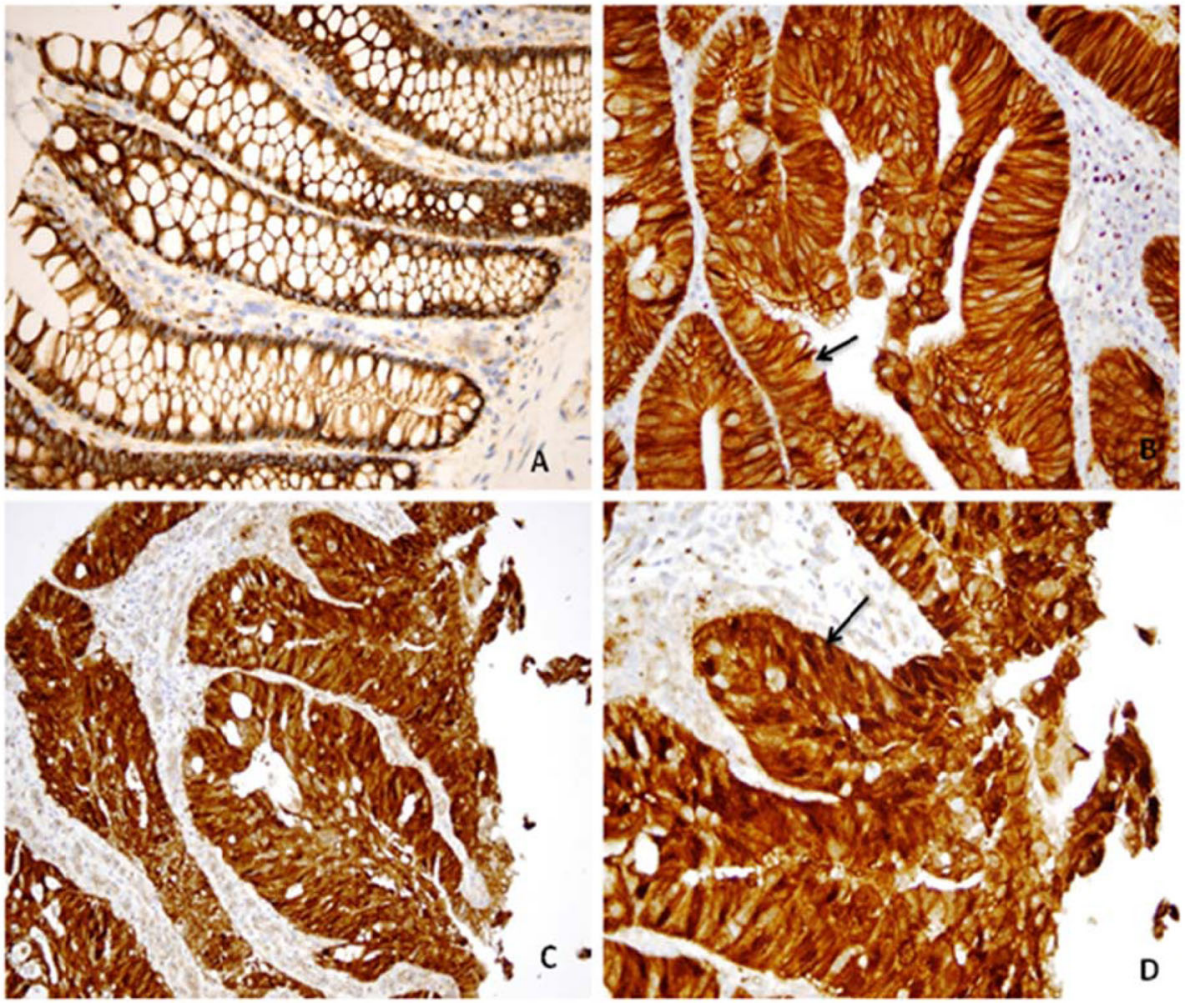
Immunostain for β-catenin in three individuals; normal (**a**, × 400), advanced adenoma (**b**, × 400) and cancer (**c** & **d**, × 200 and × 400 respectively) showing membranous staining in the normal, cytoplasmic and membranous staining in adenoma with no evidence of nuclear expression (arrow showing lack of nuclear staining) and nuclear and cytoplasmic staining in cancer (arrow showing nuclear staining)

The constructed TMAs were stained for β-Catenin. The immunostaining was carried out as follows. Dako Monoclonal Mouse Anti-Human Beta-Catenin (β-CateninC-1) intended for laboratory application to identify qualitatively by light microscopy β-catenin positive cells in normal and neoplastic tissues, was used at a dilution of 1:200, using the EnVision+, DAB (code K4006) detection system. The deparaffinized TMAs were treated prior to the IHC staining procedure. Target retrieval involved immersion of tissue sections in a pre-heated buffer solution and maintaining heat in a water bath (95–99 °C). For greater adherence of tissue sections to glass slides, silanized slides (Dako code S3003) were used. Target Retrieval Solution (code S1700) or 10x Concentrate (code S1699) using a 20-min heating protocol was used. The cellular staining pattern of anti-β-Catenin is mainly membranous, especially at the cell-cell boundaries. Positive nuclear staining and diffuse cytoplasmic staining are also reported in cancer cells (Fig. 1).

### Evaluation and assessment of the β-catenin expression

Two pathologists interpreted the IHC slides. β-Catenin expression status was assessed based on the pattern of staining (nuclear, cytoplasmic, and membranous), intensity (0 to 4 +) and percentage of staining (0 to 100%). The staining would be considered negative if there was weak or no nuclear expression, or positive if there was moderate or strong nuclear expression.

### Statistical analysis

Distribution of continuous and categorical variables were tested by Kruskal-Wallis and Chi-square test between different groups, respectively. We used logistic regression analysis to test association between the staining and risk of CRC, after adjusting for age and gender. Area under the curve (AUC) was calculated for variables with significant association with CRC using Receiver Operative Characteristics curve. All statistical analyses were performed by STATA 13.0 (STATACorp., College Station, TX).

## Results

### Epidemiological characteristics and BMI in normal, advanced adenoma and CRC

The BMI was calculated for individual patients and normal subjects as represented in Table 1. CRC patients were older (*p* = 0.004), while our healthy normal population was mostly overweight. Higher BMI was more closely associated with advanced adenoma and CRC. However, the differences were not significant (Table 1).

### Advanced adenomas and CRCs were associated with positive nuclear CTNNB1

**Fig. 2:**
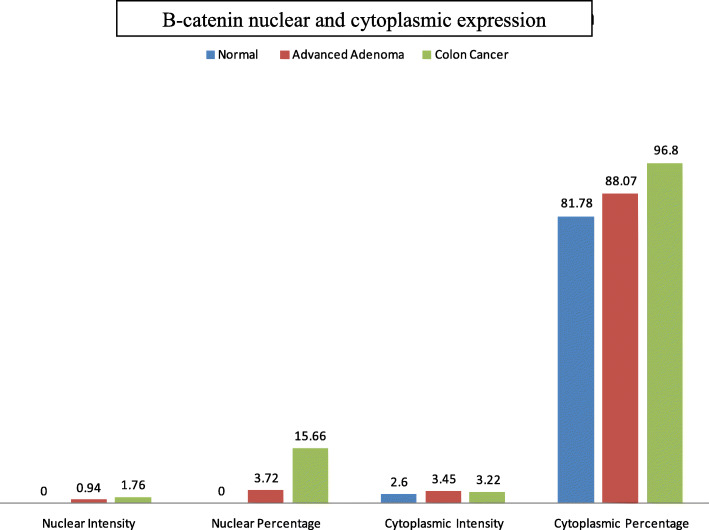
β-catenin nuclear and cytoplasmic expression in normal, advanced adenoma and CRC

We assessed whether alterations in WNT-CTNNB1 (β-Catenin) signaling plays any role colon carcinogenesis. Positive β-Catenin nuclear stains were seen in normal, advanced adenomas and CRCs were 0, 24 and 41%, respectively (*P* < 0.001; Table 2). Based on the designation of “N intensity +”, which is associated with higher risk of cancer, CRCs were associated with positive status for nuclear CTNNB1 intensity (age, gender adjusted OR: 3.40, 95%CI = 1.42–8.15, *P* = 0.006 for positive nuclear staining) compared to non-CRC samples (Normal or advanced adenoma) (Fig. 1). Nuclear staining percentage has also a fair diagnostic ability for CRC with an AUC of 0.63 (95%CI = 0.55–0.71; Table 2, Fig. 2).

**Table 2:** β-Catenin nuclear and cytoplasmic expression tabulated as intensity and percentage in normal, advanced adenoma, and CRC

	Normal *N* = 32	Advanced Adenoma *N* = 74	CRC *N* = 46	Overall *P* value	*P* value for Advanced Adenoma vs. Normal	*P* value for CRC vs. other
**C%**	100 (10–100)	100 (80–100)	100 (100–100)	0.004	0.3	0.07
**N%**	0 (0–0)	0 (0–0)	0 (0–10)	0.009	0.006	0.012
**C Intensity +**	26 (81%)	74 (100%)	46 (100%)	< 0.001	< 0.001	0.1
**N Intensity +**	0	18 (24%)	19 (41%)	< 0.001	0.002	0.001

*C* Cytoplasmic, *N* Nuclear

C Intensity+ and N Intensity+ mean Intensity 1 and above

### Overweight and obese patients show a trend with positive nuclear CTNNB1 expression

Positive nuclear CTNNB1 staining was 17% in normal weight and 27% in overweight/obese (BMI > 25) patients. This difference pointed to trend that was not statistically significant.

### Association between nuclear intensity and CRC in normal and overweight patients

The association between nuclear intensity and CRC was not statistically significant different between normal weight and overweight patients (P for interaction = 0.6; Tables 3 and 4). The positive nuclear CTNNB1 status in CRC stage III and IV (35% of all CRC) was not different from stage I and II (50% vs. 36%, respectively, *P* = 0.4).

**Table 3:** Association of BMI with β-Catenin nuclear intensity in advanced adenoma cases

Advanced adenoma with β-Catenin expression 4+ (*n* = 9) in intensity and no nuclear intensity (*n* = 28).			
	Nuclear intensity (negative) *n* = 28	Nuclear intensity (4+) *n* = 9	*P* value
BMI, median (interquartile)	29.2 (24.3–34.9)	33.2 (26.6–37.0)	0.3
Overweight, n (%)	20 (71%)	8 (89%)	0.3

**Table 4:** Association of BMI with β-Catenin nuclear intensity in CRC cases

CRC with β-Catenin expression with high nuclear intensity (4+) and without (negative).			
	Nuclear intensity (negative) *n* = 14	Nuclear intensity (4+) *n* = 12	*P* value
BMI, median (interquartile)	29.3 (18.2–40.0)	30.1 (22.8–35.3)	0.8
Overweight, *n* (%)	8 (57)	9 (75)	0.3

## Discussion

One of the important risk factors in colorectal cancer is obesity [3, 4]. β-Catenin is an E-cadherin binding protein that mediates cell-cell adhesion [20] and plays a role in the canonical WNT signaling pathway that controls the coordinated expansion and differentiation of the intestinal crypt stem cells [21]. Degradation of β-Catenin by phosphorylation followed by alteration of destruction complex (APC, GSK-3β and AXIN) results in inactivation if WNT pathway [22]. In our study, we found that was associated with an increased adjusted OR of 3.40 (95%CI = 1.42–8.15, *P* = 0.006 for positive nuclear staining) compared to non-CRC samples (Normal or advanced adenoma).

The gatekeeper gene APC is a negative regulator of β-Catenin and is mutated in approximately 80% of sporadic and hereditary colon cancers [23]. There are several mutations that can cause an accumulation of β-Catenin in tumor cells such as mutations of the APC gene, point mutations in GSK-3β or mutations in β-Catenin gene itself [23–25].

Our positive nuclear staining in CRC (41%) and its association with the positive status for nuclear CTNNB1 intensity compared to non-CRC samples are in contrast to a study by Brabletz et al. [26], which showed that β-Catenin is localized in the cytoplasm and membrane of the tumor cells whereas in our study it was mainly concentrated in the cytoplasm and the nucleus. They also mentioned that there was positive nuclear staining at the invasive front as β-Catenin is involved in tumor progression. Such is not the case in our study, indeed even when considering nuclear staining in our specimens, there was no statistically significant differences between stage III/IV cases’ staining versus stages I/II CRC cases levels of staining. The fact that β-Catenin is expressed early in the African American specimens analyzed here might partially explain the aggressive nature of CRC in this population. In addition, we showed that there is uniform membranous staining in normal and increasing cytoplasmic and nuclear staining in advanced adenomas and CRCs. This confirms that the decrease in membranous staining begins with dysplastic changes leading to a progressive disappearance at the membrane level in CRCs.

As we mentioned above, a major risk factor for CRC is obesity, which continues to expand as a pandemic worldwide [27]. The American Cancer Society Cancer Prevention Study II, states that there is an increased incidence of CRC, esophageal adenocarcinoma and other cancers with obesity [28]. In our study, we showed that 78% of advanced adenoma patients and 69% of CRC patients were overweight with BMI > 25. In comparison to advanced adenoma, the percentage was lower in cancer; perhaps due to the late stage of cancer and weight loss in the interim (Table 1). There are several mechanisms by which obesity is believed to promote CRC, this includes increase in leptin levels that cause an increase in growth and proliferation of colon cancer cells [29], altered adipokine levels, altered gut microbiome apart from increased steroid hormones and growth factors [30]. Insulin is however the established biochemical link and the main pathway involved is PIK3/AKT/mTOR pathway. Elevated IGF-1 and insulin act through the insulin receptors and phosphotidylinositide-3 kinase [31].

In addition to the above findings, we also found that overweight and obese patients (BMI > 25) did not show a significantly increased expression (*p* = 0.3) of nuclear CTNNB1 (17% positive in normal weight vs. 27% positive in overweight/obese). Morikawa et al. found that in obese patients, nuclear CTNNB1 positivity was associated with significantly better cancer-specific survival suggesting that WNT signaling acts as a switch and when it is on, adipogenesis is repressed. Kennell et al. demonstrated that activated Frz1 (frizzled) promotes β-Catenin stability, inhibits apoptosis, and adipogenesis. Ross, et al. also showed that Wnt signaling acts as a molecular switch that controls adipogenesis. Upregulation of Wnt signaling maintains preadipocytes in an undifferentiated state and when Wnt signaling is prevented they differentiate into adipocytes [17, 32, 33].

**Fig. 3:**
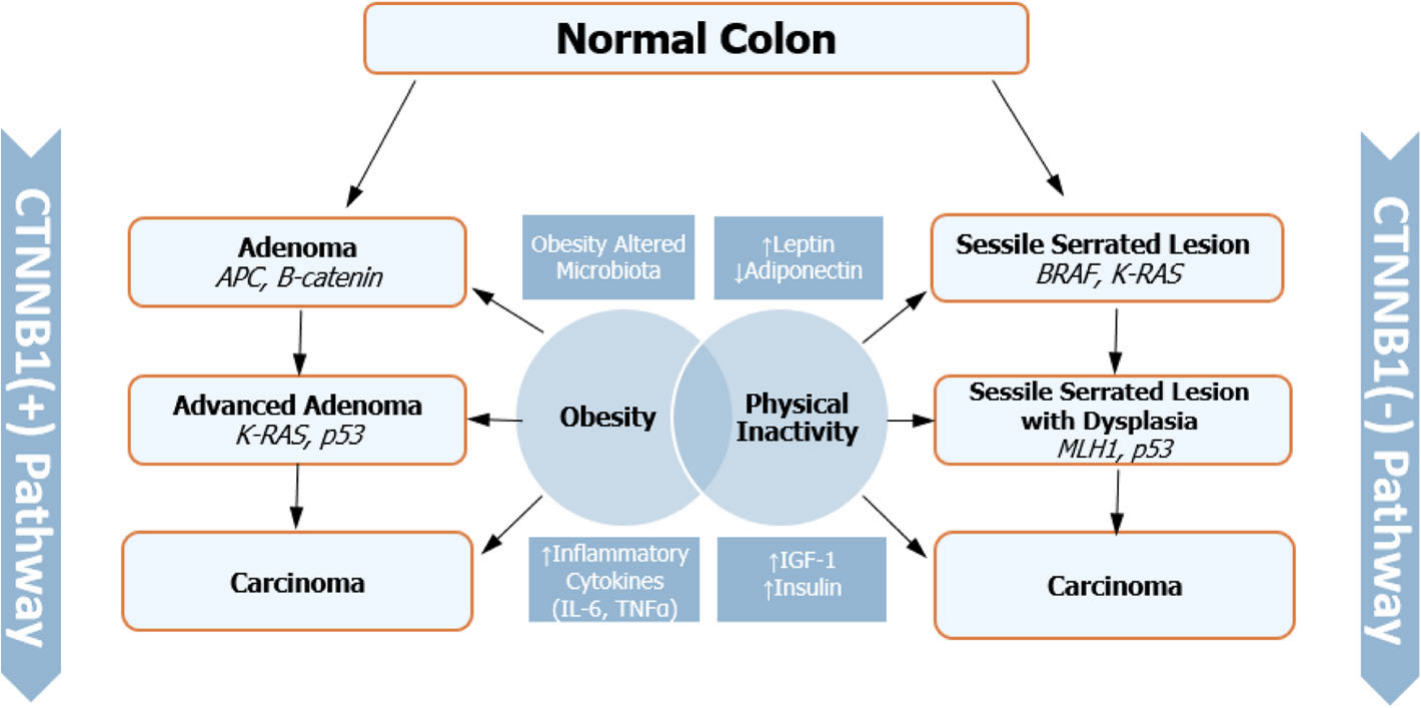
The putative relationship between obesity and colorectal cancer evolution pathways by cellular CTNNB1 status, based on the data by the current study. Our study suggests that there is no association between obesity and CTNNB1 expression

Although in our study there was no association between nuclear intensity and CRC between normal and overweight patients (P for interaction = 0.6), there is accumulating evidence to show that the state of chronic inflammation incited by obesity might play a role in promoting colorectal carcinogenesis [8, 34]. Of the many markers, TNF-α is important [35, 36], as it activates WNT signaling through the induction of GSK-3β phosphorylation, resulting in increased nuclear localization of β-Catenin [37]. In addition to TNF-α, other humoral agents associated with obesity might also be contributing to the activation of WNT signaling like IL-1β and adiponectin, which is decreased in the obese state and is not an inflammatory cytokine that can modulate GSK3β/β-Catenin signaling pathway [38]. Although multiple mechanisms may be operating in parallel and contributing to the pro-tumorigenic milieu, Wnt is a pivotal tumorigenic pathway [39], aberrations of which is important in the evolution of most sporadic CRC. In summary, there is positive nuclear staining in CRCs (41%), which was associated with the positive status for nuclear CTNNB1 intensity (adjusted OR: 3.40, 95%CI = 1.42–8.15%, *P* = 0.006 for positive nuclear staining) compared to non-CRC samples (Normal or advanced adenoma). This shows that advanced adenomas and CRCs were associated with activation of β-Catenin in physically fit, overweight and obese patients (Fig. 3).

## Conclusion

Advanced adenoma and CRC were associated with activation of β-Catenin in physically fit, overweight and obese patients. Thus, participation of obesity and WNT pathway seem to be independent CRC factors in African American patients. Inflammation-driven activation of WNT signaling as a potential pathway linking obesity to the development of CRC needs to be investigated further in the African American population. This might provide insights into the identification of new therapeutic targets to reduce the burden of obesity-associated CRC.

## Data Availability

All data generated or analyzed during this study are included in this published article.

## Abbreviations

CRC: Colorectal cancer
WAT: White adipose tissue
AA: African Americans
